# Challenge of treating skeletal muscle metastasis during the COVID-19 pandemic in a low-resource setting

**DOI:** 10.3332/ecancer.2021.1235

**Published:** 2021-05-18

**Authors:** Maria Gloria Elisha Casas, Mamer Rosario, Geoffrey Battad, Adrienne Camille Mercado, Trisha Ann Hermogenes, Alvin Hernandez, Janelyn Dy-Ledesma, Avelino Alomesen, Juancho Lorenzo Valera, Arnel Christian Dy

**Affiliations:** 1 Department of Surgery, University of the East Ramon Magsaysay Memorial Medical Center (UERMMMC), Doa Imelda, Quezon City 1113, Philippines; 2 Department of Orthopaedics, East Avenue Medical Center, Diliman, Quezon City 1101, Philippines; 3 Department of Pathology, UERMMMC, Doa Imelda, Quezon City 1113, Philippines; 4 Department of Pathology, East Avenue Medical Center, Diliman, Quezon City 1101, Philippines

**Keywords:** lung cancer, COVID-19, skeletal muscle metastasis, biopsy, sarcoma, frozen autograft reconstruction

## Abstract

**Background:**

The authors report on an extremely rare case of skeletal muscle metastasis from primary lung cancer that involved the radial nerve and humerus, which was ‘over-treated’ with wide tumour resection and frozen autograft reconstruction upon misdiagnosis of sarcoma by intraoperative frozen section, amid pressure of expediting hospital care in a low-resource setting during the coronavirus disease (COVID-19) pandemic.

**Case presentation:**

A 61-year-old male living outside Metro Manila presented with painful mass in his left distal arm during the enhanced community quarantine, and requested admission upon testing negative for COVID-19. Imaging studies suggested a diagnosis of soft tissue sarcoma involving the radial nerve and humerus, and intending to prevent nosocomial severe acute respiratory syndrome coronavirus 2infection of patient, treatment was expedited by foregoing biopsy and opting for intraoperative frozen section prior to resection. Frozen section findings suggested malignancy intraoperatively, and surgical team proceeded with wide tumour resection and frozen autograft reconstruction of the humerus using plates and screws. However, permanent sections revealed metastatic carcinoma from primary non-small cell lung cancer, with positron emission tomography (PET) scan confirming lung mass in the right apical lobe.

**Conclusion:**

The report concludes that establishment of a definite tumour diagnosis by final histopathological analysis is indispensable, even when planning for emergent surgery in the time of the COVID-19 pandemic.

## Introduction

Lung carcinoma is the leading cause of cancer-related deaths worldwide, with about half of cases already metastatic at the time of diagnosis [1]. Soft tissue metastases affecting skeletal muscle, subcutaneous tissue and skin are rarely reported in the literature [2], but perceived as sign of advanced disease and considered a grave prognostic indicator [3]. The prevalence of skeletal muscle metastasis (SMM) with any carcinoma ranges from 0.8% to 17.5% [4], although studies in lung cancer patients found a lower prevalence ranging from 0% to 0.8% [4]. The extreme rarity of SMM must be explained by the apparent resistance of skeletal muscle to metastatic disease, as postulated by the mechanical, metabolic and immunologic hypotheses [5]. Although prior confirmation by biopsy is essential for SMM’s prognostic implications, SMM can be macroscopically indistinguishable from other soft tissue tumours [3, 4], as well as several benign diseases [13]. More importantly, the differential diagnosis must be posed with primary soft tissue sarcomas [14], since SMM from primary carcinoma of the lung is extremely rare and ominous.

The severe acute respiratory syndrome coronavirus 2 (SARS-CoV-2) has spread to many countries worldwide, with the WHO declaring a coronavirus disease (COVID-19) pandemic on 11 March 2020 [6]. The resilience of many hospitals has been tested, with healthcare systems basically caught unprepared for the scale of the pandemic [7]. Healthcare systems were forced to adapt due to rising numbers of active COVID-19 disease, with some suspending hospital admissions and elective surgeries for non-COVID-19 cases to allow for surges of COVID-19 patients and prevent nosocomial spread of the SARS-CoV-2 [8]. Early studies into the pandemic have found cancer patients especially vulnerable to SARS-CoV-2 infection [9, 10], while the recent collaborative study by 235 institutions in 24 different countries on SARS-CoV-2 infected patients undergoing surgery has found cancer diagnosis a predictor of perioperative mortality [11]. Healthcare workers must face the challenge of preventing nosocomial COVID-19 disease among cancer patients needing urgent care in a hospital setting. Delaying surgery for stable tumours, changing chemotherapy timing and schedule and avoiding frequent hospital visits were precautions recommended to avoid viral nosocomial spread for cancer patients in general [12]. Approaching cases of SMM can therefore be very challenging in the time of COVID-19 pandemic, as tumour surgeons were placed under immense pressure by the rising number of active SARS-CoV-2 infections and the need to avoid exposure of uninfected cancer patients. Making decisions under pressure can be unfortunate when basic principles of cancer treatment, especially warranting a low threshold for biopsy, are sacrificed in the process. We hereby report on a case of SMM from primary lung cancer that involved the radial nerve and distal humerus which we ‘overtreated’ by performing wide tumour resection [15] and frozen autograft reconstruction [16], upon wrongly presuming a frozen section diagnosis of sarcoma with the aim of expediting the surgical care in a low-resource setting, during the COVID-19 pandemic.

## Case presentation

**Figure 1.**
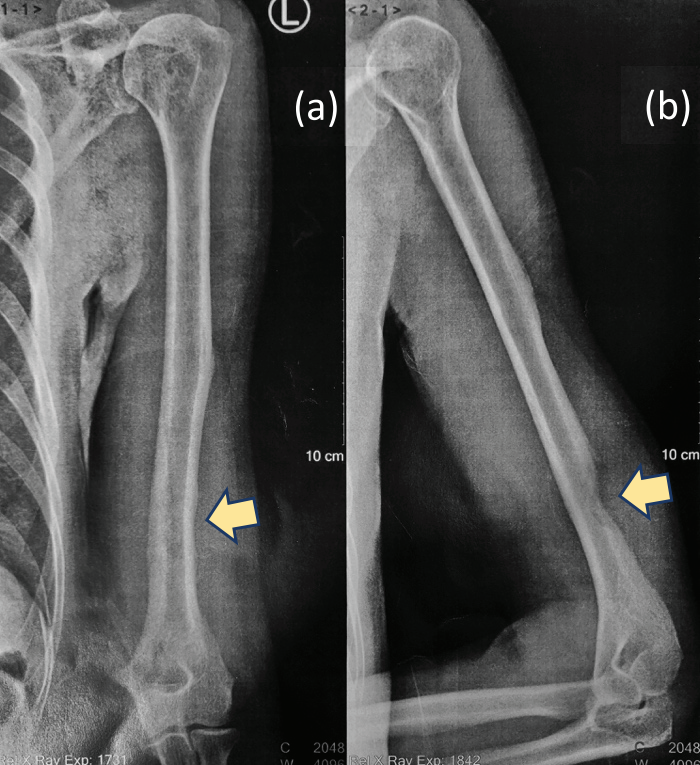
(a): Anteroposterior and (b): lateral radiographs of the left arm showing erosion of the posterolateral cortex of the distal humerus (yellow arrows).

**Figure 2.**
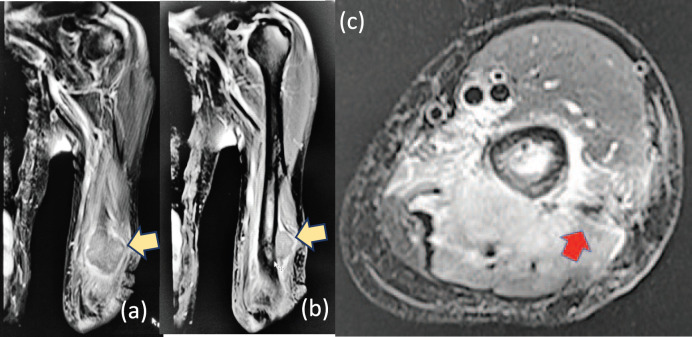
T2-weighted (a): coronal, (b): sagittal and (c): axial MRI confirming a heterogeneously enhancing intramuscular mass involving the triceps (yellow arrows), measuring 3 cm × 4 cm × 4.5 cm associated with cortical erosion of posterolateral humerus and partial tumoural encasement of the radial nerve (red arrow).

**Figure 3.**
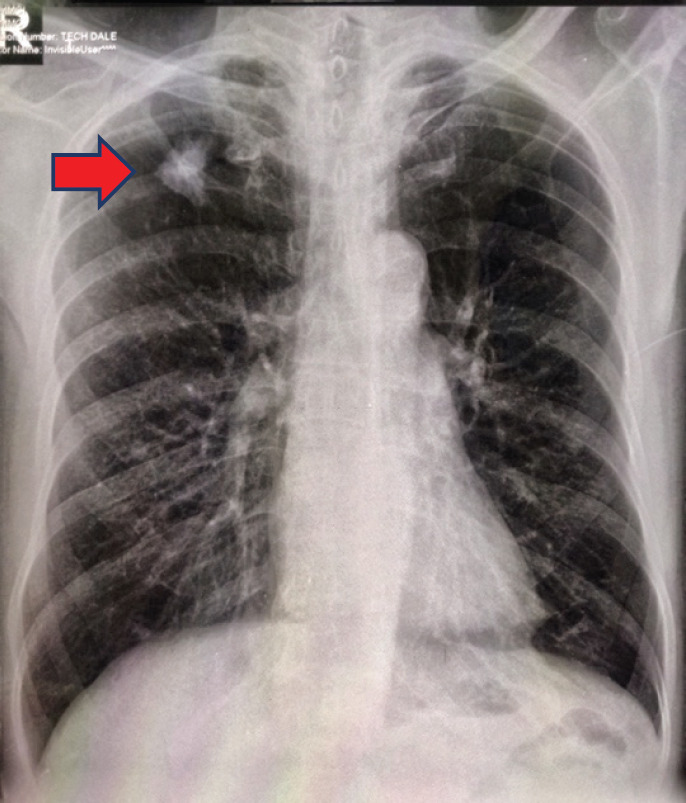
Chest radiography showing ill-defined infiltrates in the right upper lobe (red arrow).

A 61-year-old male from Lucena City (located 134 km south of Manila), right-hand dominant with 20 pack-year smoking history, presented with a painful soft tissue lump on the posterolateral aspect of his left distal arm with no other accompanying symptoms, at our institution in Metro Manila in May 2020 when the entire island of Luzon was under a state of enhanced community quarantine (ECQ). Our institution then catered to both patients with and without COVID-19. The patient underwent core-needle biopsy at another hospital a month prior to consult, revealing fibrocollagenous tissue with nonspecific chronic inflammation on final histopathology. On physical examination, there were no neurological deficits, although Tinel’s sign was positive. Review of left arm radiographs done at another hospital shows cortical erosion of the distal humerus with periosteal elevation (Figure 1a and b), while magnetic resonance imaging (MRI) confirmed a heterogeneously enhancing intramuscular mass involving the triceps measuring 3 cm × 4 cm × 4.5 cm (Figure 2a), associated with cortical destruction of, and abnormal marrow signals in the posterolateral humerus (Figure 2b), and partial tumoural encasement of the radial nerve (Figure 2c). Our differential diagnoses included benign and malignant primary soft tissue tumours, but upon review of chest radiographs done at another hospital, we noted finding of heterogenous ill-defined opacity in the right upper lobe that was officially read as tuberculosis (Figure 3). Although pulmonary tuberculosis was a possibility, we were then highly considering presence of sarcomatous metastasis in the lung. Financial constraints prevented further evaluation by chest computed tomography (CT).

During the ECQ, travel was restricted for most people, and many cities controlled their borders in an attempt to contain local transmission of COVID-19. The patient tested negative for COVID-19 by reverse transcriptase polymerase chain reaction (PCR) testing and therefore, requested to be admitted for continuation of care. But for fear of getting nosocomial SARS-CoV-2 infection, he pleaded for a short inpatient stay and an expedited hospital care. Citing early reports having found cancer patients especially vulnerable to SARS-CoV-2 infection [9, 10], and cancer diagnosis a predictor of perioperative mortality [11], we agreed to forego performing preoperative biopsy. Imaging findings demonstrating aggressive biology and the possibility of pulmonary metastasis added up to a primary consideration of soft tissue sarcoma for our case. Thus, without benefit of a biopsy-proven diagnosis, our team decided to request for intraoperative frozen section to rule out malignancy, and prepare for wide tumour resection with frozen autograft reconstruction of the humerus. Without advantages of having an active cancer tumour board in the interim, we adopted recommendation by the French Sarcoma Group [17] to pursue surgery even for high grade soft tissue sarcomas with distant metastasis in operable patients without COVID-19 symptoms.

**Figure 4.**
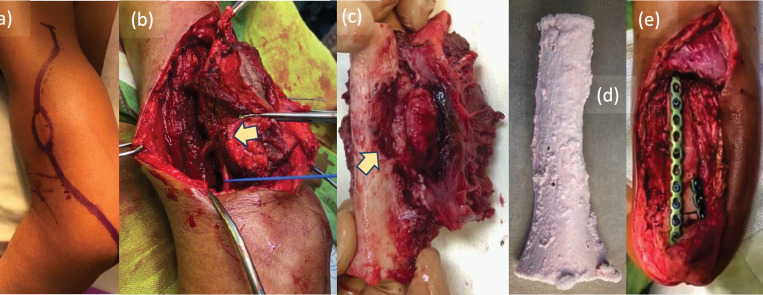
Intraoperative photos. (a): Tumour approached via posterolateral incision to include biopsy site, (b): with radial nerve (blue line) seen adherent to tumour (yellow arrow) during wide resection. (c): Resected tumour-bearing bone seen eroded (yellow arrow) by the soft tissue mass. (d): Resected bone free of tumoural tissues treated with liquid nitrogen using free-freezing method, then thawed, washed with distilled water and (e): fixed to humerus using plates and screws.

**Figure 5.**
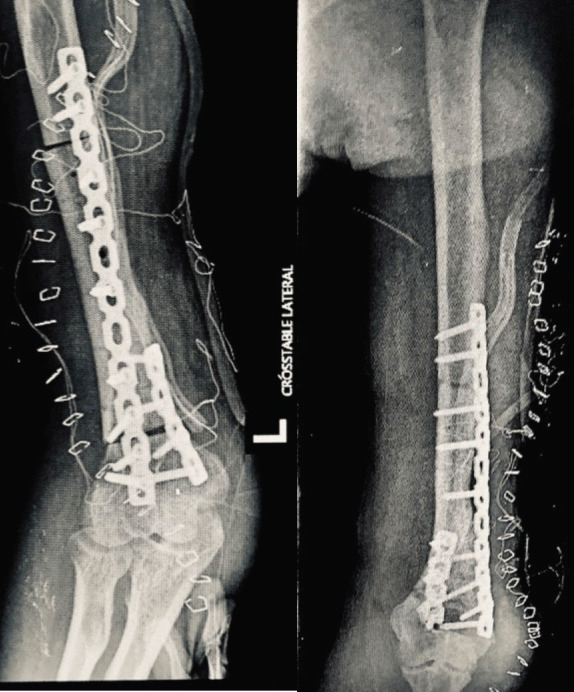
Postoperative (a): anteroposterior and (b): oblique radiographs of the left arm showing stable reconstruction of the humerus with acceptable alignment.

The surgery was performed under general anaesthetic, with members of the surgical team additionally wearing Tyvek® coveralls (DuPont™; Delaware, USA), N95 respirators (3M™; Minnesota, USA) and face masks. We approached the tumour posterolaterally (Figure 4a), with the patient on right lateral decubitus position. Prior to resection of the tumour, tumoural tissue was obtained and sent intraoperatively for histopathological analysis, revealing signs of malignancy by frozen section. We therefore ruled in soft tissue sarcoma, and decided to resect the tumour with wide margins. Upon dissection, radial nerve was adherent to tumour and cortical bone was visibly eroded (Figure 4b). Wide bone margins were identified by C-arm guidance, and humeral segment together with involved radial nerve was included in the resection (Figure 4c). Resected tumour-bearing bone was freed of tumoural tissues then soaked in liquid nitrogen (Figure 4d), thawed, washed with distilled water and re-applied to humerus by fixation using plates and screws (Figure 4e). The operation lasted 361 minutes from induction to skin closure with no intraoperative complications, and estimated blood loss was 300 mL. After the surgery, patient’s left elbow was immobilised with arm sling, and postoperative arm radiographs showed stable humeral reconstruction with acceptable alignment (Figure 5a and b). We strategised to start physical therapy upon notice of bridging callus by radiographic surveillance, and patient was discharged well with unremarkable inpatient course.

**Figure 6.**
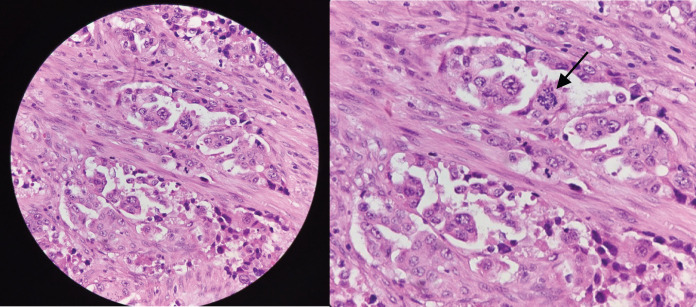
(a): Routine histologic section of tumour revealing malignant epithelial cells disposed in nests and exhibiting ill-formed glandular lumen and (b): plump epithelial cells with moderate cytoplasm seen on high-power view exhibiting nuclear atypia (black arrow), anisocytosis and prominence of nucleoli.

Final histopathological findings of the operative sample, with immunohistochemistry study, concluded the diagnosis of metastasis of an adenocarcinoma of lung origin (Figure 6a and b). The resection margins were negative for tumoural involvement. Positron emission tomography scan was done, which showed a lobulated, calcified lung mass within the right apical segment measuring 2.4 cm × 1.9 cm × 3.2 cm, as well as two rim-enhancing nodules in both frontal lobes suggestive of brain metastases. Patient was then referred to medical and radiation oncology services for palliative treatment, and at the time of writing, has completed ten sessions of whole-brain radiation therapy at another hospital and is to start adjuvant pemetrexed (Alimta®; Eli Lilly and Co.; Indiana, USA) and carboplatin (Biovinate®; Unilab, Inc.; Mandaluyong City, PH) treatment. Physical therapy is also done regularly on outpatient basis.

## Discussion

The first case of COVID-19 in the Philippines was reported last 20 January 2020, although its first local transmission in the country was only confirmed on 7 March 2020 [18]. The Philippine government then placed the entire island of Luzon under ECQ, by which travel was restricted for most people except for frontline medical and security personnel, and persons delivering essential goods and services [19]. Cities were forced to close their borders in an attempt to contain the local transmission, and the ECQ was further expanded nationwide. In a country where cancer treatment centres are concentrated in highly-urbanised areas, the ECQ undeniably made access to cancer care more difficult for patients living in far-off provinces due to the unavailability of public transportation and limitation of drug availability [20].

A mass is palpable in about 78% of cases of soft tissue metastases but pain is the most frequent symptom, involving approximately 83% of cases [21]. A painful, palpable soft tissue lump in the posterolateral aspect of the patient’s left arm prompted consult at the emergency department, despite an ECQ that required crossing multiple border controls from 134 km south of the hospital, and period when clinic visits were suspended to help prevent nosocomial spread of the SARS-CoV-2. A key challenge to cancer care during the pandemic is uncertainty, and the anxiety that arises from it [20]. Upon request by the patient to have shorter hospital stay and recognition of his fear of getting nosocomial COVID-19, the need to expedite hospital care without the advantage of a biopsy-proven diagnosis became a crucial dilemma in our case.

Although clinical features of SMM can mimic those of soft tissue sarcoma, a painful mass is more commonly observed in patients with SMM [22]. However, the absence of other symptoms, in addition to erosion of the adjacent bone with marrow infiltration seen on plain radiography and MRI, made us highly consider the possibility of sarcoma in our case. Despite the presence of an ill-defined opacity in the patient’s right upper lobe on plain chest radiography, we considered, at the most, the possibility of sarcomatous metastasis and not primary lung carcinoma. Due to the extreme rarity of SMM, several authors [4, 13, 14] agree the differential diagnosis must be posed with primary soft tissue sarcomas and muscle lymphomas, as well as benign lesions such as haemangiomas, intramuscular ganglia and myxomas. Although MRI is not specific for diagnosing SMM, it has been considered an indispensable tool for distinguishing SMM from sarcomas [23], typically showing intramuscular lesions with poorly defined margins, low signal intensity on T1-weighted sequences, high signal intensity with surrounding oedema on T2-weighted sequences and enhancement with gadolinium [24, 25]. Most soft tissue sarcomas similarly display low signal intensity on T1-weighted sequences and high signal intensity on T2-weighted sequences [26], although erosion of adjacent bone by SMM is rarely evident on MRI [25]. Moreover, MRI displayed a heterogeneously enhancing soft tissue mass in our case. Misdiagnosis of SMM is therefore possible not only due to rarity of the disease but also the variety of clinical and radiological manifestations. Although a prior biopsy was the ideal step to gather key diagnostic information that shall guide subsequent treatment, we were forced to decide against the otherwise standard tenet of performing core-needle biopsy before surgery [27], in the aim of shortening the patient’s hospital stay. It was at this point wherein we wrongly considered a working impression of sarcoma, even if only the histologic examination allows the definite diagnosis. Upon discussing all concerns with the patient and his family, we ultimately decided on performing frozen section during the surgery, with our strategy for tumour resection dependent on whether findings were suggestive of malignancy. We came across proposals by the French Sarcoma Group for management of sarcomas during the COVID-19 pandemic [17], and adopted its recommendation to pursue surgery for high grade soft tissue sarcomas in operable patients without COVID-19 symptoms, notwithstanding distant metastasis. Although studies on safety of limb salvage surgery during the COVID-19 pandemic were lacking, we prepared to perform wide tumour resection [15] and frozen autograft reconstruction [16], in case frozen section analysis turns out positive for malignancy. Nevertheless, an ill-defined lung opacity on chest radiographs of a male over 50-years old with 20 pack-year smoking history should have evoked first the diagnosis of lung cancer. Both lung fibroscopy and CT scan would have cost less than an urgent surgical operation.

Up to the time of writing, there have been no studies evaluating the diagnostic accuracy of frozen section for intraoperative pathologic diagnosis of SMM. Although intraoperative frozen section has been advocated by some authors as accurate and cost-effective method for diagnosing musculoskeletal sarcomas [28], the latter are relatively rare compared to carcinomas and present greater challenge to intraoperative frozen section consultation [29]. Moreover, studies agree that frozen section should not be used to replace paraffin embedded tissue technique even if the former can provide rapid diagnosis, since the former is still comparatively inferior to the latter due to limitations [29]. We realised that even in the time of COVID-19 pandemic, histopathology plays an integral role in the approach to treating patients with soft tissue tumours, the accuracy of which has particularly important therapeutic implications.

A review by Perisano *et al* [1] emphasises role for radiotherapy, chemotherapy or both in treating painful SMM from lung carcinoma in the context of widespread metastatic disease, although the authors stressed that palliative treatment with surgical debulking is indicated if pain and neurovascular damage are becoming clinically significant. The Philippine Society of Medical Oncology came up with guidelines to address concerns in the management of adult patients with solid tumours during the COVID-19 pandemic [19], and highlighted the need to classify conditions into stable and urgent disease based on individualised patient assessment. Cancer patients with urgent conditions, like our patient who had advanced lung cancer with symptomatic SMM eligible for palliative surgical debulking, may warrant hospital care but taking into consideration a balanced decision with the risk of the patient contracting nosocomial COVID-19 [19]. Our patient consented to a surgical treatment with the request that his hospital stay be made short as possible. But thinking our case to be possibly sarcoma upon intraoperatively documenting involvement of the radial nerve by the tumour, in addition to bone erosion seen on imaging and the verification of malignancy by frozen section, we proceeded with wide tumour resection including the involved radial nerve [15], and frozen autograft reconstruction with bicolumnar distal humeral plating [16]. We adopted the frozen autograft technique developed by the Kanazawa University in Japan to perform reconstruction following malignant bone tumour resection, using liquid nitrogen to destroy tumour cells by ice crystal formation and cell dehydration [30]. Kimura *et al* [16] recommend frozen autograft reconstruction following resection of malignant or aggressive benign tumours of the humerus, having found acceptable complication rate and functional outcomes in their series. However, we unquestionably ‘overtreated’ the case for we could have spared the radial nerve, and performed just palliative surgical debulking if we knew it was a case of SMM from primary carcinoma of the lung. Euanorasetr and Suwanthanma [31] reported on their case of infected gastric schwannoma, and could have avoided a Billroth 2 procedure if only a prior open biopsy was done that could have ruled out their preoperative working diagnosis of gastric lymphoma. Two other cases of gastric schwannoma preoperatively misdiagnosed as malignancies ended up with partial gastrectomies, instead of just local tumour extirpations [32, 33]. Although these, including our surgical procedure, were instances of ‘overtreatment’ and not ‘undertreatment’, the anatomical and corresponding functional losses from such surgeries must be undesirable for the patient. Likewise, misdiagnosing malignancy and consequently ‘undertreating’ a tumour are never wanted occurrences for the treating tumour surgeon [34].

## Conclusion

The authors conclude that establishment of a definite tumour diagnosis by final histopathological analysis is indispensable, even when planning for emergent surgery in the time of COVID-19 pandemic. We agree with recommendations to promote a less aggressive approach and delay hospital visits for those with stable disease, in the aim of preventing nosocomial SARS-CoV-2 spread for cancer patients [11, 12, 19]. The pandemic has forced us to rethink the way we approached our case, and both the speed with which the pandemic has spread and the disruption it caused on multiple aspects of our daily life have provided us little time to understand how best to care for our patient. Taken altogether, we learn that regardless of pressing times we are in, basic principles of cancer care, especially warranting a low threshold for biopsy, must never be compromised.

## Conflicts of interest

The authors declare that they have no competing interests.

## Authors’ contributions

Both MGEC and MR wrote the initial draft of the manuscript. GB, ACM, TAH, AH, JDL, AA, JLV and ACD reviewed the manuscript and were involved in its critical revision before submission. All authors read and approved the final manuscript.

## Ethics approval and consent to participate

This article has been approved by the Ethics Review Committee of the UERMMMC Research Institute for Health Sciences (reference code: 0883/H/2020/111). Consent to participate was obtained from the patient and his family.

## Funding statement

The authors had no funding sources for this study.
